# 16-membered ring macrolides and erythromycin induce *ermB* expression by different mechanisms

**DOI:** 10.1186/s12866-022-02565-3

**Published:** 2022-06-09

**Authors:** Weizhi He, Kai Jiang, Hua Qiu, Lijun Liao, Shasha Wang

**Affiliations:** 1 Fudan University Shanghai Cancer Center, Institutes of Biomedical Sciences, Shanghai Key Laboratory of Medical Epigenetics, International Co-laboratory of Medical Epigenetics and Metabolism (Ministry of Science and Technology), Shanghai Medical College of Fudan University, Shanghai, 200032 China; 2 Department of Biobank, Renji Hospital, School of Medicine, Shanghai JiaoTong University, Shanghai, China; 3 Department of General Surgery, The First Affiliated Hospital of Nanchang University, No.17 Yongwai Zheng Street, Nanchang, 330006 Jiangxi Province China; 4 Department of Anesthesiology and Pain Management, Shanghai East Hospital, School of Medicine, Tongji University, Shanghai, 200120 China

**Keywords:** Translation arrest, Ribosome stalling, 16-membered ring macrolides, Erythromycin, Spiramycin, Tylosin, Multidrug resistance

## Abstract

**Background:**

Ribosome stalling on *ermBL* at the tenth codon (Asp) and mRNA stabilization are believed to be mechanisms by which erythromycin (Ery) induces *ermB* expression. Expression of *ermB* is also induced by 16-membered ring macrolides (tylosin, josamycin and spiramycin), but the mechanism underlying this induction is unknown.

**Methods:**

We introduced premature termination codons, alanine-scanning mutagenesis and amino acid mutations in *ermBL* and *ermBL2.*

**Results:**

In this paper, we demonstrated that 16-membered ring macrolides can induce *ermB* expression but not *ermC* expression. The truncated mutants of the *ermB*-coding sequence indicate that the regulatory regions of *ermB* whose expression is induced by Ery and 16-membered ring macrolides are different. We proved that translation of the N-terminal region of *ermBL* is key for the induction of *ermB* expression by Ery, spiramycin (Spi) and tylosin (Tyl). We also demonstrated that *ermBL*2 is critical for the induction of *ermB* expression by erythromycin but not by 16-membered ring macrolides.

**Conclusions:**

The translation of *ermBL* and the RNA sequence of the C-terminus of *ermBL* are critical for the induction of *ermB* expression by Spi and Tyl.

**Supplementary Information:**

The online version contains supplementary material available at 10.1186/s12866-022-02565-3.

## Background

Macrolides have been used clinically for over 70 years. These antibiotics inhibit Gram-positive and several Gram-negative bacteria [1]. These antibiotics exert their therapeutic effects by antagonizing the growth of bacteria via the inhibition of protein synthesis by narrowing the nascent protein exit tunnel (NPET) in ribosomes [2–4]. However, macrolide antibiotics can also induce the expression of several resistance genes [5–8]. For example, macrolide antibiotics promote ribosome stalling on the regulatory leader peptide *ermCL* or *ermBL* and then induce the expression of *ermC* or *ermB* [6, 7, 9]. The *ermC* and *ermB* genes encode a ribosomal methylase that dimethylates a single adenine in 23S rRNA, dramatically reduces the affinity of macrolides for the ribosome, causing a high level of macrolide resistance and cell survival [10, 11]. The rapid emergence of drug resistance in bacterial pathogens makes many antibiotics, including macrolide antibiotics, ineffective. In response to this threat, other nonclinical large-scale antibiotics may be substitutes for clinical use. Till date, 16-membered ring macrolides are rarely used in the clinic; therefore, there is no large-scale emergence of bacteria that are resistant to 16-membered ring macrolides in the clinic. Such antibiotics can be used as a potential alternative approaches for treatment in the clinic. According to traditional understanding, 16-membered ring macrolides can’t induce the expression of resistance genes [12]. With increasing research, it was found that 16-membered ring macrolides could also induce the expression of resistance genes. Yakhin et al. showed that ribosome stalling at an RYR arrest motif in the C-terminus of a leader peptide found upstream of *yxjB* (encodes an enzyme that methylates 23S rRNA) is believed to be the mechanism by which tylsoin induces *yxjB* expression [13]. All macrolide-lincosamide-streptogramin B (MLS_B_) antibiotics, including 16-membered ring macrolides, act as inducers of *ermB* expression to various degrees [10, 14]. However, the mechanism by which 16-membered ring macrolides induce *ermB* expression is not well known. We wanted to study how 16-membered ring macrolides induce the expression of *ermB*. The results of this study may avoid the large-scale emergence of drug-resistant bacteria in the clinical use of 16-membered macrolides.

The expression of *ermB* can be either constitutive (M19270) [15] or inducible (M11180) [16] depending on the regulatory region located upstream of the *ermB* gene. The regulatory region includes a short leader peptide, which is also called *ermBL*, with its own ribosome binding site (RBS1), a nontranslational loop-stem structure, several *ermB* (*ermB’*) coding sequences, and its own ribosome binding site (RBS2). In addition, in our previous study, we also found that another leader peptide named *ermBL*2 is present in the regulatory region and is critical for erythromycin (Ery)-mediated induction of gene expression (Fig. S1) [17].

When the expression of *ermB* is inducible, ribosome stalling and mRNA stability are believed to control its expression [6, 18, 19]. In the *Enterococcus faecalis* strain DS16 transposon Tn917 (M11180) [16], *ermB* is preceded by a 258 nucleotide leader region, which contains two regulatory open reading frames, *ermBL*, which encodes a 27 amino acid-long leader peptide, and *ermBL2*, which encodes a 16 amino acid-long leader peptide [6, 17, 19, 20]. This regulatory region has been well studied in previous research [6, 19, 20]. Ribosome stalling induced by erythromycin takes place on the tenth codon (Asp) of *ermBL* and induces a conformational switch in the mRNA, which exposes the ribosome binding site (RBS2) of *ermB* to the ribosome; then, *ermB* is translated (Fig. S1).

In addition to erythromycin, the expression of *ermB* is also induced by 16-membered ring macrolides (tylosin, josamycin, and spiramycin) [10, 21]. However, two groups have demonstrated that ribosome stalling on the tenth codon (Asp) of *ermBL* by 16-membered ring macrolides has not been observed [6, 22, 23]. Mutations in the *ermBL* region exerted differential effects on the induction of gene expression by 14- and 16-membered ring macrolides [14, 21], suggesting that different mechanisms by which 16-membered ring macrolides induce *ermB* expression may exist.

Fourteen and fifteen-membered ring macrolides have been effective in the clinic for many years. Therefore, there are a large number of drug-resistant bacterial species that are resistant to these antibiotics in the clinic. To date, over 40 published *erm* genes, constitutive or inducible, have been identified in bacteria, and inducible *erm* expression has been shown to be induced by 14- and 15-membered ring macrolide antibiotics. 16-membered macrolide antibiotics include a disaccharide at position C-5. They are mainly used in veterinary medicine and are rarely used in the clinic, and there is no large-scale emergence of drug-resistant bacteria in the clinic. Notably, 16-membered macrolides are generally reported not to induce the expression of inducible *erm* genes. Studying the mechanism by which 16-membered macrolides induce the expression of *ermB* is conducive to formulating more reasonable strategies of drug use and significantly delaying the emergence of drug-resistant bacteria. Therefore, our results provide important insights into the clinical potential of these underexplored 16-membered ring macrolide antibiotics for use against drug-resistant human pathogens. The study of the mechanism underlying inducible drug resistance is helpful for treatment and for the prevention of the emergence of strains resistant to 16-membered ring macrolide antibiotics.

## Materials and methods

### Antibiotics, enzymes, chemicals and growth conditions

The antibiotics (erythromycin, spiramycin, and tylosin) were obtained from Sigma–Aldrich. Isopropyl-β-D-thiogalactopyranoside (IPTG) and 5-bromo-4-chloro-3-indolyl-D-galactopyranoside (X-Gal) were purchased from Sigma–Aldrich. Luria–Bertani (LB) broth components and agar were purchased from Sangon Biotech Co., Ltd. (Shanghai). The restriction endonuclease used for DNA cloning was obtained from Fermentas. All the oligonucleotide primers were synthesized by Sangon Biotech (Shanghai) Co., Ltd. Site-directed mutagenesis was performed with a QuikChange® Site-Directed Mutagenesis Kit (Stratagene). *E. coli* strains were grown in Luria–Bertani broth (LB) at 37 °C unless noted for different applications.

### Bacterial strains and plasmids

The plasmid pGEX-4T-3 (GE Healthcare) was used as the vector for the generation of the pGEX-*ermBL*-*ermB’*-*lacZ*α reporter plasmid as described in a previous study [17]. pGEX-*ermCL*-*ermC’*-*lacZ*α was constructed in this study. The pGEX-*ermCL*-*ermC’*-*lacZ*α reporter plasmid has the same sequence as pGEX-*ermBL*-*ermB’*-*lacZ*α, except that the *ermCL*-*ermC’* sequence was replaced with the *ermBL*-*ermB’* sequence*. The ermCL*-*ermC’* sequence was shown in a previous study [24, 25]. All the cloning procedures and most experiments with the engineered constructs were carried out with *E. coli* strain JM109 (Promega) [*endA1, recA1, gyrA96, thi, hsdR17 (rk–, mk* +*), elA1, supE44, Δ (lac-proAB), [F' traD36, proAB, laqIqZΔM15*].

### Construction of the pGEX reporter plasmid

The pGEX reporter plasmid was constructed as described in a previous study [17]. In short, the pGEX vector replaced some new multiple cloning site sequences (SmaI-KpnI-XbaI-AflII-XhoI-TthIIII) with sequences between BspMI and TthIIII. The *ermCL-ermC’* cassette from *Staphylococcus aureus* plasmid pE194 (X03097) [25] included the tac promoter, the leader ORF and part of the *ermC* coding sequence (*ermC’*). The *ermC-ermC’* cassette was cloned between the XbaI site and AflII site of the pGEX vector to produce the translational fusion plasmid pGEX-Ptac-*ermCL*-*ermC’*. *ErmC’* is the N-terminus of the *ermC* gene. *ErmC’* has no ribosomal methylase activity but is essential for conformational changes in the proposed model of the induction of *ermC* expression by erythromycin [7]. In this paper, we used *ErmC’* as ten amino acids of the N-terminus of *ErmC.* The reporter gene *lacZα* was cloned into a vector following *ermC’* with AflII and XhoI.

### Disc diffusion assay of pGEX reporter activation

The disc diffusion assay protocol was carried out as previously described [24]. Briefly, JM109 cells transformed with the pGEX reporter plasmid were shaken overnight in LB broth supplemented with 100 μg/mL ampicillin at 37 °C. The culture was diluted 1:100 in fresh LB broth supplemented with ampicillin (100 μg/mL) and IPTG (0.5 mM) and shaken at 37 °C until the OD_600_ approached 0.2–0.5. Then, we mixed the cells into 8 ml of 0.8% LB agar at 50 °C. After brief mixing, the cell suspension was poured on top of a 1.5% LB agar plate (10 cm dish) supplemented with 100 μg/mL ampicillin, 0.5 mM IPTG, and 160 μg/ml X-Gal. After the soft agar had solidified, discs were placed on agar, and the appropriate antibiotic was added. The plates were incubated for 18 to 24 h at 37 °C. The disc diffusion experiment used in our study aimed to qualitatively analyze gene expression induction by antibiotics. The intensity of the color of the blue ring relative to the blue background of plate, which reflects the extent of induction, was qualitatively assessed. The concentrations of antibiotics used were as follows: Ery (64 mg/ml), Spi (128 mg/ml), Tyl (128 mg/ml) and Chl (64 mg/ml).

### β-galactosidase assay

The β-galactosidase assay method was used as described previously [17]. *E. coli* strains carrying the pGEX reporter plasmid were grown in LB until OD600 ≈ 0.2. Cultures were split and treated with a series of concentrations of antibiotics. The cultures were incubated at 37 °C for 1.5 h with shaking at 220 rpm. The OD600 of the resuspended cells was measured. The cells were centrifuged and then thoroughly suspended with 1 ml Z buffer (60 mM Na_2_HPO4, 40 mM NaH_2_PO4, 10 mM KCl, 1 mM MgSO_4_, pH 7.0) and shaken vigorously to lyse the cells by the addition of 100 μl chloroform and 50 μl 0.1% SDS. The assays were performed at 28 °C with 200 μl ONPG (O-nitrophenol-β-D-galactopyranoside, 4 mg/ml, Sigma). The reaction was stopped after a sufficient yellow color developed by adding 0.5 mL of 1 M Na_2_CO_3_. Then, the samples were centrifuged and monitored at 420 nm. β-Galactosidase activities were calculated in Miller Units using the following formula: β-Galactosidase activity = A420 × 1000 × min^–1^ × ml^–1^ × A600^–1 ^[26]. At least three independent biological replicates were performed. Unless otherwise specified, the concentrations of antibiotics used were as follows: Ery (256 μg/ml), Spi (1024 μg/ml) and Tyl (1024 μg/ml).

## Results

### 16-membered macrolides could specifically induce the expression of *ermB*

**Fig. 1:**
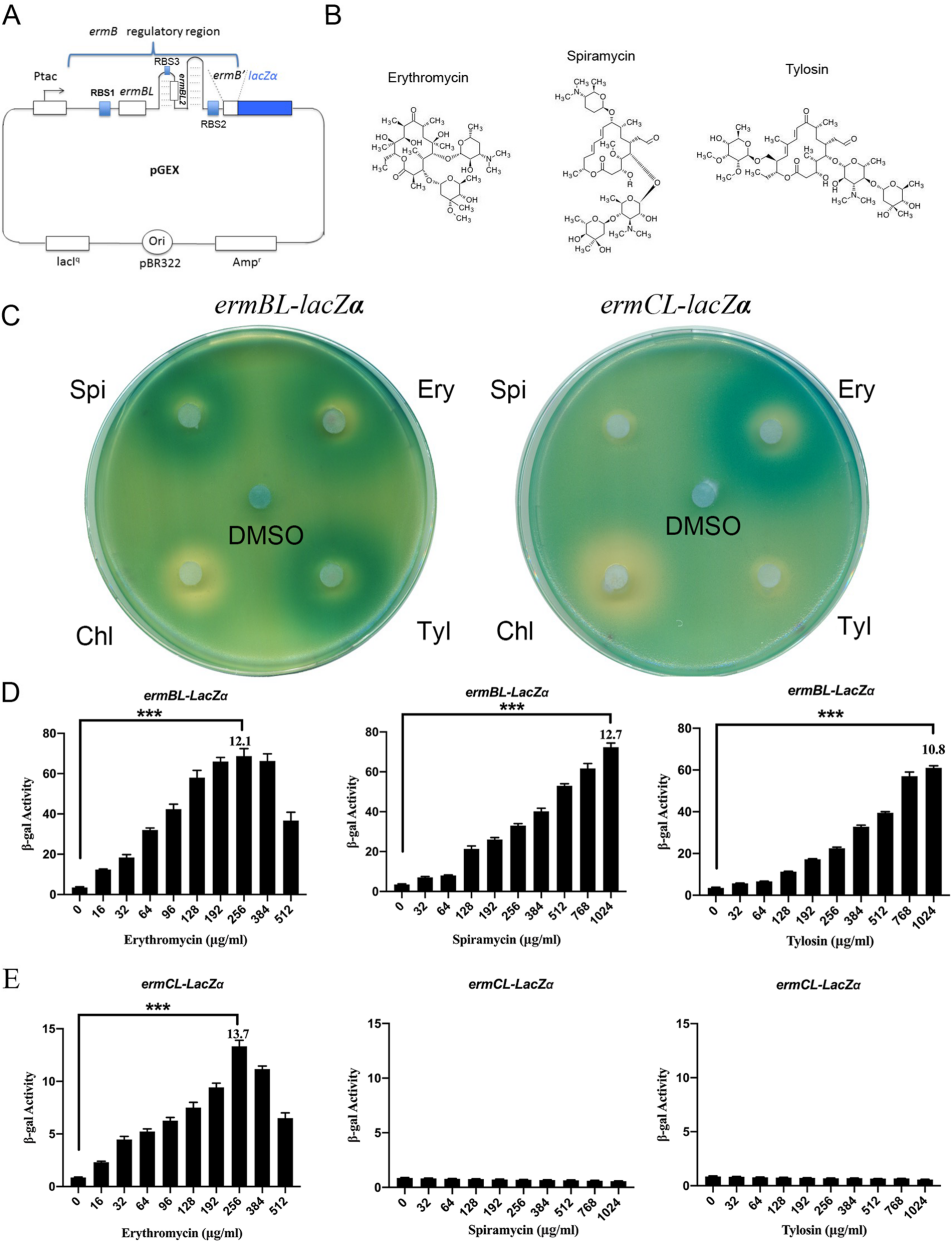
16-membered ring macrolide antibiotics induce *ermB* expression. **A** The structure of the *ermB*-based pGEX reporter plasmid. **B** Chemical structures of the macrolide antibiotics used in this work. **C** Agar diffusion assays of cells transformed with the reporter plasmid containing the *ermBL-lacZα* or *ermCL-lacZα* reporter grown on plates supplemented with IPTG and X-gal; each filter disc was spotted with erythromycin (Ery), spiramycin (Spi), tylosin (Tyl), chloramphenicol (Chl) and DMSO. **D-E** β-Galactosidase activity assays of the *ermBL* or *ermCL-lacZα* reporter gene on titration of erythromycin, spiramycin and tylosin. Miller units of β-galactosidase activity are shown on the Y-axis. The different concentrations of antibiotics are shown on the X-axis. The error bars correspond to the SEM of three independent experiments. The number on the top of each bar represents the largest fold change in beta-gal activity between the antibiotic and DMSO group. ***P < 0.001; (unpaired two-tailed Student’s t test)

In a previous study, we constructed a pGEX-*ermBL*-*ermB’*-*lacZ*α reporter plasmid that has a tac promoter (Ptac) following a regulatory region *ermBL-ermB’* operon and translational fusion with *lacZα* (Fig. 1A, S2) [17]. We also constructed a new pGEX-*ermCL*-*ermC’*-*lacZ*α reporter plasmid in this study. This reporter system allows for the easy monitoring of the induction of gene expression by antibiotics, either by measuring β-galactosidase enzyme activity (quantitative analysis) or by observing X-Gal hydrolysis on plates using a disc diffusion assay (qualitative analysis). Erythromycin is a 14-membered ring macrolide, while spiramycin and tylosin are 16-membered ring macrolides (Fig. 1B). Tylosin and spiramycin are 16-membered macrolide antibiotics that include a disaccharide at position C-5, with tylosin also bearing a mycinose substituent at position C-23.

In a previous study, *ermB* expression was found to be strongly induced by subinhibitory concentrations (approximately 25% MIC) of Ery, Spi and Tyl [10, 18]. We first determined the minimal inhibitory concentration (MIC) of antibiotics for *E. coli* carrying the pGEX-*ermBL*-*ermB’*-reporter plasmid (Table S1). We next used our plasmid to qualitatively and quantitatively investigate the difference in reporter activation by a number of antibiotics at subinhibitory concentrations. Ery, Spi and Tyl activated the *ermBL*- *ermB’* reporter at a broad range of concentrations (Fig. 1C, D). As expected, chloramphenicol (Chl) and the antibiotic solvent DMSO could not induce *ermB* expression (Fig. 1C). These results showed that Ery, Spi and Tyl induced the expression of *ermB*. In a previous study, a well-studied model of *ermCL* expression from the *Staphylococcus aureus* plasmid pE194 revealed that *ermCL* expression was also strongly induced by erythromycin but not spiramycin or tylosin [24, 27–29]. As expected, in our reporter system, Ery but not Spi and Tyl could induce the expression of *ermC* (Fig. 1C, E). *ErmC* expression could not be induced by 16-membered ring macrolide antibiotics or chloramphenicol (Fig. 1C, E). In summary, Ery can induce the expression of *ermB* and *ermC,* while 16-membered ring macrolides can specifically induce the expression of *ermB.*

### *ErmBL* translation is key for the induction of *ermB* expression by 16-membered ring macrolides

**Fig. 2:**
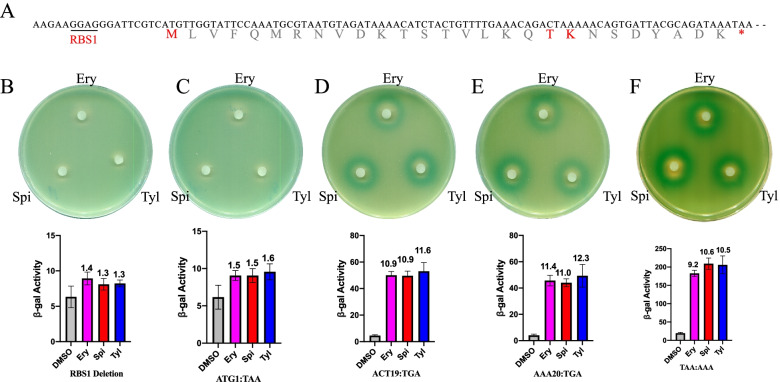
Translation of the N-terminus of *ermBL* is critical for the induction of *ermB* expression by 16-membered ring macrolides. **A** The detailed RNA and amino acid sequence of *ermBL* and its own ribosome binding site (RBS1). **B-E** β-Galactosidase activity assay and disc diffusion assay of various *ermBL* mutants exposed to erythromycin, spiramycin and tylosin. Miller units of β-galactosidase activity are shown on the Y-axis. The number on the top of each bar represents the fold change in beta-gal activity between the antibiotic and DMSO group. The error bars correspond to the SEM of three independent experiments. All the β-Galactosidase activity of antibiotics group compared to DMSO group is significantly. *P* < 0.05; (unpaired two-tailed Student’s t test)

Ribosome stalling on the tenth codon of *ermBL* is believed to be the major mechanism by which erythromycin induces *ermB* expression [6, 20]. One hypothesis suggested that small ORFs located in the 5’ untranslated region may act as cis mRNA stabilizers, increasing the half-life of the downstream transcripts [30, 31]. To evaluate whether *ermBL* translation is critical for the induction of *ermB* expression by Spi and Tyl, we used various constructs in the regulatory region of *ermBL* described in a previous study (Fig. 2A) [17]. We found that Ery, Spi and Tyl could no longer induce the expression of downstream genes with RBS1 (GGAGGG) deletion (Fig. 2B). We also found that the induction of expression by Ery, Spi and Tyl was severely impaired when the start codon of *ermBL* was mutated to a stop codon (ATG to TAA) (Fig. 2C), which means that *ermBL* expression is critical for the induction of *ermB* expression by Ery, Spi and Tyl. When we changed the 19th codon of *ermBL* to a stop codon (ACT19:TGA) and used the previously constructed plasmid with the 20th codon of *ermBL* mutated to a stop codon (AAA20:TGA) [17], the C-terminus of *ermBL* contained premature termination. *ermB* expression could also be induced by Ery, Spi and Tyl, which means that the translation of the N-terminus of *ermBL* is necessary for the induction of *ermB* expression by Ery, Spi and Tyl, while translation of the C-terminus (K20-K27) of *ermBL* is not important (Fig. 2D, E). The leader ORF *ermBL* encodes 27 long amino acids, and we changed the stop codon TAA to AAA, so *ermBL* will encode a 30-amino acid long peptide. Even though the length of *ermBL* was changed, induction of its expression still occurred, which shows that the length of the regulation leader peptide does not affect the induction of *ermB* expression by Ery, Spi and Tyl (Fig. 2F). Taken together, these results verify that *ermBL* translation is key for the induction of *ermB* expression by 16-membered ring macrolides.

### Induction of *ermB* expression by Ery and 16-membered ring macrolides occurs via different mechanisms

**Fig. 3:**
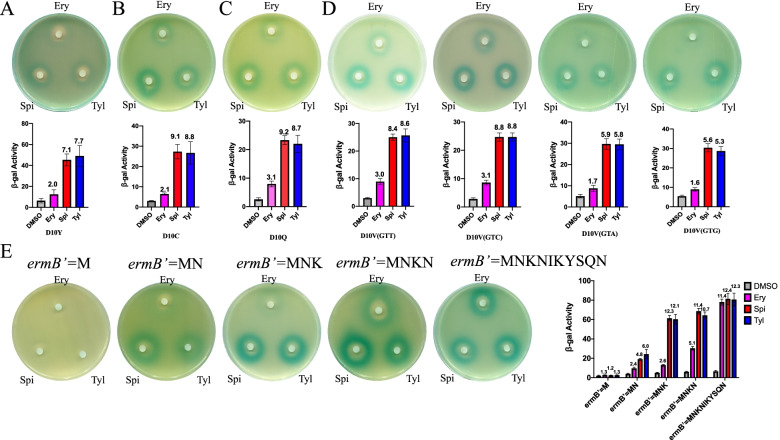
The mechanisms by which erythromycin and 16-membered ring macrolides induce *ermB* expression are different. **A-D** Mutation of the tenth codon of *ermBL* to other amino acids. β-Galactosidase activity and disk diffusion assays of the activation of the *lacZα* reporter in response to Ery and 16-membered ring macrolides in vivo. Miller units of β-galactosidase activity are shown on the Y-axis. The number on the top of each bar represents the fold change in beta-gal activity between the antibiotic and DMSO group. The error bars correspond to the SEM of three independent experiments. All the β-Galactosidase activity of antibiotics group compared to DMSO group is significantly. *P* < 0.05; (unpaired two-tailed Student’s t test). **B** β-Galactosidase activity assay and disc diffusion assay of *ermB*’ truncated mutants exposed to erythromycin, spiramycin and tylosin. Miller units of β-galactosidase activity are shown on the Y-axis. The number on the top of each bar represents the fold change in beta-gal activity between the antibiotic and DMSO group. The error bars correspond to the SEM of three independent experiments. All the β-Galactosidase activity of antibiotics group compared to DMSO group is significantly. *P* < 0.05; (unpaired two-tailed Student’s t test)

The tenth codon Asp of *ermBL* is a key amino acid for ribosome stalling, and ribosome stalling in the tenth codon is critical for the induction of *ermB* expression by Ery [6, 18]. In previous study, several groups showed that ribosome stalling in the tenth codon of *ermBL* could not be observed by in vitro toe-printing [6, 22, 23]. To investigate whether the tenth codon Asp of *ermBL* is a key amino acid for the induction of *ermB* expression by 16-membered ring macrolides, we used reporter constructs that had been constructed in our previous study in which the tenth codon was mutated to other amino acids [17]. Gupta et al. showed that replacement of Asp10 with tyrosine, cysteine, glutamine or valine abolished Ery-mediated translation arrest at codon 10 [19]. When the tenth codon Asp of *ermBL* was mutated to these amino acids that affect Ery-specific ribosome stalling in vitro, we wondered whether the induction of *ermB* expression by Ery, Spi and Tyl was affected in vivo*.* When the Asp10 codon of *ermBL* was replaced with tyrosine, cysteine, glutamine or valine, the induction became Spi- and Tyl-dependent, while the induction of reporter gene expression by Ery was abolished (Fig. 3A, B, C, D), indicating that the mechanisms underlying the induction of gene expression by Ery and 16-membered ring macrolides are different. To evaluate whether the RNA sequence of *ermBL* is important as an amino acid, we changed the Asp10 codon to valine encoded by different codons (GTT, GTC, GTA, GTG). The β-galactosidase assay and disc diffusion assay showed that induction of reporter gene expression by Ery was severely impaired by all the D10V mutations, while the reporter gene expression was also induced by Spi and Tyl (Fig. 3D), indicating that the difference between Ery and 16-membered ring macrolides was not RNA sequence dependent.

Gupta1 et al. showed that the induction of *ermB* expression by Ery and telithromhycin could be affected by single amino acid changes in the *ermBL* sequence that switch the specificity of recognition of distinct antibiotics [19]. To verify whether the different mechanisms by which Ery and 16-membered ring macrolides induce *ermB* expression are also caused by the different antibiotics recognizing different amino acids in *ermBL*, we made mutations in the *ermB* regulatory region but not in *ermBL*. In a proposed classic model of mechanisms underlying induction of gene expression, the N-terminus of *ermB* (*ermB*’) also contributed to the change in secondary structure (Fig. S1). We generated truncated mutations of *ermB*’ (Fig. S2) and surprisingly found that the regions required for the induction of expression by these two types of antibiotics were different (Fig. 3E). In this paper, when *ermB*’ equals the first  ten amino acids of the N-terminus of *ermB*, we found that reporter gene expression could be well induced by Ery, Spi and Tyl (Fig. 1C, B, D, E). When *ermB’* contains only one amino acid (methionine), *ermB* expression could not be induced by these two kinds of antibiotics. When *ermB’* has two amino acids (MN), *ermB* expression could be moderately induced by 16-membered ring macrolides but not by Ery. When *ermB’* includes three amino acids (MNK), *ermB* expression could be completely induced by 16-membered ring macrolides but not Ery. When *ermB’* has four amino acids (MNKN), *ermB* expression could be completely induced by 16-membered ring macrolides and moderately induced by Ery. The *ermB* regulatory region that is necessary for the induction of *ermB* expression by Ery and 16-membered ring macrolides is different. In summary, we showed that 16-membered ring macrolides and Ery induce the expression of *ermB* via different mechanisms, which is not because of different stalling efficiencies due to one amino acid change in the leader peptide sequence.

### Alanine-scanning mutagenesis of *ermBL* confirms that 16-membered ring macrolides and Ery induce *ermB* expression via different mechanisms

**Fig. 4:**
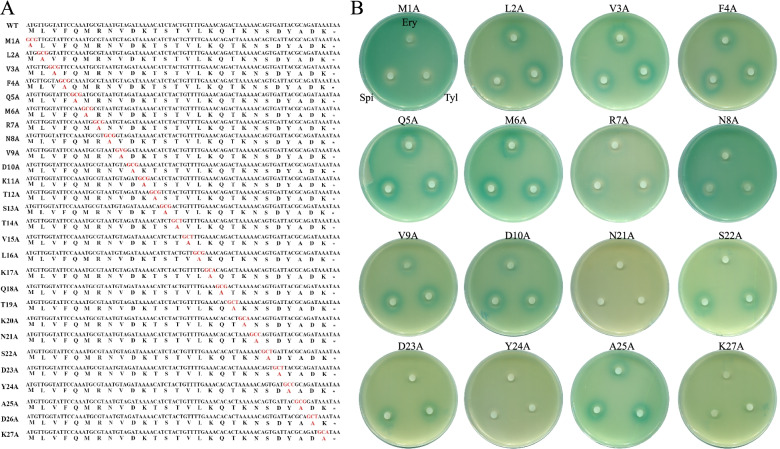
Alanine-scanning mutagenesis of *ermBL*. **A** The amino acid sequence of *ermBL* peptide (WT) and its alanine-scanning mutagenesis. **B** Disc diffusion assay of various *ermBL* alanine-scanning mutagenesis

In a previous study, the seventh codon to eleventh codon of *ermBL* (R7-K11) were found to be key amino acids for the translation arrest induced by erythromycin [6, 22]. Our data showed that *ermBL* translation is critical for the induction of *ermB* expression, while Asp10 is not important for the induction of *ermB* expression by 16-membered ring macrolides. To distinguish which amino acids of *ermBL* are necessary for the induction of *ermB* expression by 16-membered ring macrolides, we used an alanine-scanning mutagenesis assay (Fig. 4A). Similar to the previous conclusion, the M1A mutation disrupts the translation of *ermBL*, so the induction of *ermB* expression by Ery, Spi and Tyl is impaired (Fig. 4B). L2-M6 (the second codon to the sixth codon of *ermBL*) is not important for the induction of *ermB* expression by Ery, and it is not important for the induction of *ermB* expression by 16-membered ring macrolides. In contrast to erythromycin, alanine-scanning mutagenesis of *ermBL* demonstrated that R7-K11 (the seventh codon to the eleventh codon of *ermBL*) is not critical for the induction of *ermB* expression by spiramycin and tylosin because the induction of *ermB* expression by spiramycin and tylosin is less impaired than that by erythromycin once these key amino acids are mutated to alanine (Fig. 4B). Unexpectedly, we found that the C-terminus of *ermBL* (N21, Y24, K27) is critical for the induction of *ermB* expression by 16-membered ring macrolides (Fig. 4B) (Fig. S4)**.** However, Fig. 2 shows that the translation of the last codons of *ermBL* is not critical for the induction of *ermB* expression by 16-membered ring macrolides. These contradictory results indicate that the C-terminus of *ermBL* is crucial for antibiotic-induced *ermB* expression, not because it is the amino acid of *ermBL*, but for other reasons. When we changed an amino acid in the the C-terminus of *ermBL* to alanine (Fig. 4B), we changed not only the amino acid sequence but also the RNA sequence of the C-terminus of *ermBL.* Figure 2 shows that the amino acids of the last codons of *ermBL* are not critical for the induction of *ermB* expression by 16-membered ring macrolides. Therefore, we have reason to think that the RNA sequence, not the amino acid sequence, of the C-terminus of *ermBL,* is critical for the induction of *ermB* expression by 16-membered ring macrolides.

In our previous work, we showed that the last sequence of *ermBL* (N21-K27) function as N-terminus of *ermBL2* are very important for Ery-mediated induction of *ermB* expression [17]. However, S22A and D23A mutations maintained the induction of *ermB* expression by 16-membered ring macrolides, but these mutations reduced the effect of Ery, which further shows that the two kinds of antibiotics have different mechanisms by which they induce *ermB* expression.

### The latter part of the *ermB* regulatory region is a key component for the induction of *ermB* expression by spiramycin and tylosin

To determine which region of *ermBL* is key for the induction of *ermB* expression by spiramycin and tylosin, we engineered several hybrid leaders using the feature that 16-membered ring macrolides induce *ermB* expression rather than *ermC* expression. This difference provides a good model for investigating the mechanism by which 16-membered ring macrolides induce *ermB* expression.

**Fig. 5:**
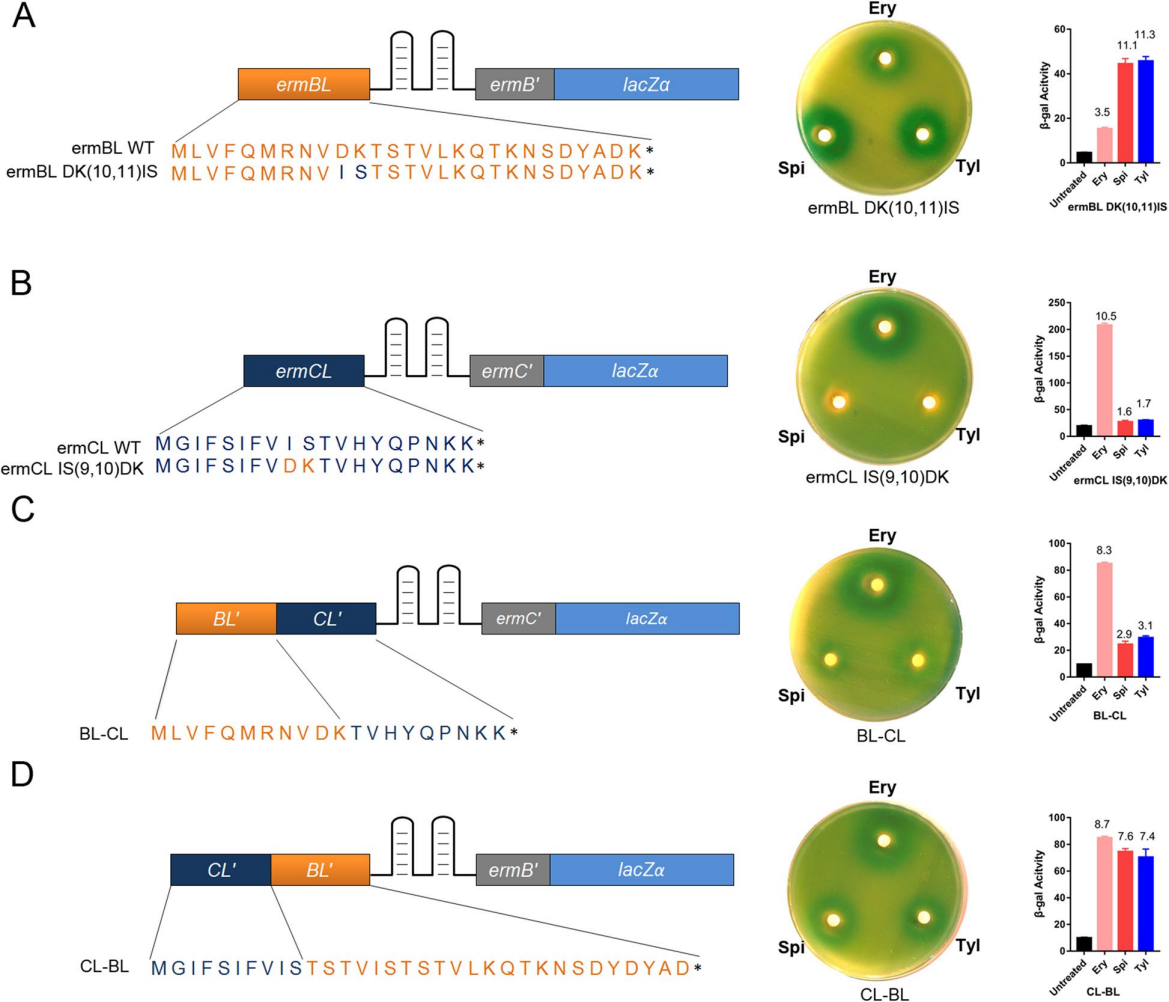
The latter part of the *ermB* regulatory region is key for the induction of *ermB* expression by spiramycin and tylosin. **A** Codons 10(Asp) and 11(Lys) of WT *ermBL* were mutated to isoleucine and serine, respectively, to mimic the identified P- and A-site codons in ribosomes stalled at *ermCL. ***B** Codons 9 (isoleucine) and 10 (serine) of WT *ermCL* were mutated to aspartic acid and lysine, respectively, to mimic the identified P- and A-site codons in ribosomes stalled at *ermBL. ***C** The first part of the ERY-inducible *ermCL-lacZα* reporter plasmid is a substitute for *ErmBL*-controlled ribosome stalling (M1-K11), named BL-CL. **D** The first part of the ERY-inducible *ermBL-lacZα* reporter plasmid is a substitute for *ErmCL*-controlled ribosome stalling (M1-S10), named CL-BL. β-Galactosidase activity assay and disc diffusion assay of these mutants exposed to erythromycin, spiramycin and tylosin. Miller units of β-galactosidase activity are shown on the Y-axis. The number on the top of each bar represents the fold change in beta-gal activity between the antibiotic and DMSO group. The error bars correspond to the SEM of three independent experiments. All the β-Galactosidase activity of antibiotics group compared to DMSO group is significantly. *P* < 0.05; (unpaired two-tailed Student’s t test)

Codons 9 (isoleucine, I) and 10 (serine, S) of WT *ermCL* are P- and A-site codons in ribosomes that are stalled at *ermCL* by erythromycin, while Codons 10 (asparagine, D) and 11 (lysine, K) of WT *ermBL* are P- and A-site codons in ribosomes that are stalled at *ermBL* by erythromycin [6, 7]. We mutated the Asp10 and Lys11 codons of *ermBL* to Ile and Ser, respectively (*ermBL* DK [10, 11] IS). We found that it had no effect on the induction of *ermB* expression by sipramycin and tylosin (Fig. 5A), but the induction by erythromycin was impaired (Fig. 5A). E*rmBL* DK [10, 11] IS changed IS codons from the ninth and tenth codons of *ermCL* to the tenth and eleventh codons of *ermBL*, and changing the length of the nascent peptide dramatically reduced the efficiency of stalling in IS, as shown in a previous study [7]. We also mutated the ninth (I) and tenth (S) codons of *ermCL* to Asp and Lys, respectively (*ermCL* IS [9, 10] DK), and we found that the expression of *ermC* was not induced by spiramycin and tylosin as *ermCL* WT (Fig. 5B, 1C, and E). Taken together, the results suggest that D10-K11, which is key for the induction of *ermB* expression by erythromycin, is not a key amino acid for the induction of *ermB* expression by spiramycin and tylosin.

To distinguish which part is key for the induction of *ermB* expression by spiramycin and tylosin, we tailored the ERY-inducible *ermCL-lacZa* reporter fusion to the *ErmBL*-controlled ribosome stalling part (M1-K11) named BL-CL (Fig. 5C) (Fig. S3). We found that spiramycin and tylosin did not induce the expression of the gene in this fusion plasmid (Fig. 5C). This result illustrated that the first part of *ermBL* does not determine the induction of expression by 16-membered ring macrolides. We also tailored the ERY-inducible *ermBL*-lacZa reporter fusion to the *ermCL*-controlled ribosome stalling part (M1-S10) named CL-BL (Fig. 5D) (Fig. S3) and found that spiramycin and tylosin induced gene expression similar to erythromycin (Fig. 5D). In summary, these data showed that the latter part of the *ermB* regulatory region (T12 of *ermBL* to *ermB*’) is key for the induction of expression by spiramycin and tylosin.

### Translation of *ermBL2* is not critical for the induction of *ermB* expression by spiramycin and tylosin

**Fig. 6:**
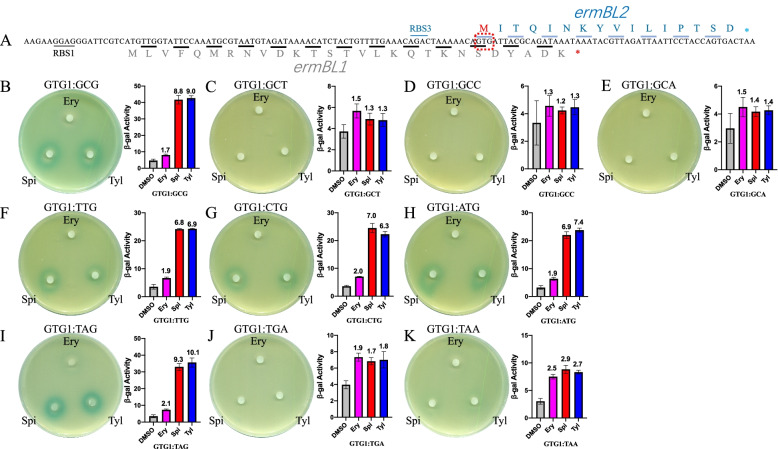
*ermBL2* is not critical for the induction of *ermB* expression by spiramycin and tylosin. **A** The detailed RNA and amino acid sequences of *ermBL*, *ermBL*2 and their own ribosome binding sites (RBS3). **B-E** Mutation of the start codon (GTG) of *ermBL2* to alanine (GCG, GCT, GCC, GCA). β-Galactosidase activity and disk diffusion assays of the activation of the *lacZα* reporter in response to Ery and 16-membered ring macrolides in vivo. **F–H** Mutation of the start codon (GTG) of *ermBL2* to other start codons (ATG, CTG, TTG). β-Galactosidase activity and disk diffusion assays of the activation of the *lacZα* reporter in response to Ery and 16-membered ring macrolides in vivo. **I-K** Mutation of the start codon (GTG) of *ermBL2* to stop codons (TAG, TGA, TAA). β-Galactosidase activity and disk diffusion assays of the activation of the *lacZα* reporter in response to Ery and 16-membered ring macrolides in vivo. Miller units of β-galactosidase activity are shown on the Y-axis. The number on the top of each bar represents the fold change in beta-gal activity between the antibiotic and DMSO group. The error bars correspond to the SEM of three independent experiments. All the β-Galactosidase activity of antibiotics group compared to DMSO group is significantly. *P* < 0.05; (unpaired two-tailed Student’s t test)

The above results show that the translation of *ermBL* and the C-terminal region of er*mBL* are key for the induction of *ermB* expression by erythromycin, spiramycin and tylosin. In addition, the mechanism underlying the induction of expression by Ery and 16-membered ring macrolides is different. Additionally, the amino acid sequence of the C-terminal region of *ermBL* is not critical for *ermB* induced by spiramycin and tylosin. In our previous work, we showed that a new leader peptide, *ermBL2*, is present in the *ermB* regulatory region and is critical for the induction of *ermB* expression by Ery (Fig. 6A) [17]. Therefore, we hypothesized that the importance of the C-terminus of *ermBL* is either because the amino acid sequence of *ermBL2* or the RNA sequence itself affects the induction of *ermB* expression by 16-membered ring macrolides.

We wanted to determine whether the expression of *ermBL2* was critical for the induction of *ermB* expression by Spi and Tyl. We used previous constructs in which the start codon (GTG) of *ermBL2* was mutated to alanine (GCG, GCT, GCC, GCA) [17]. In vivo β-galactosidase assays and disc diffusion assays showed that erythromycin did not induce *ermB* expression with all alanine mutations, while spiramycin and tylosin lost the ability to induce the expression of *ermB* with most alanine mutations except the GTG1:GCG mutation (Fig. 6B, C, D, E). We also used previous constructs in which the start codon (GTG) of *ermBL2* was mutated to other stronger or weaker start codons (ATG, TTG, CTG) [17]. These single nucleotide mutations eliminated the effect of Ery on the induction of *ermB* expression while maintaining the effect of Spi and Tyl on the induction of *ermB* expression (Fig. 6F, G, H). We then used previous constructs in which the start codon of *ermBL2* (GTG) was mutated to a stop codon (TAA, TAG, TGA) [17] and found that the induction of *ermB* expression by Ery was impaired by all stop mutations, while spiramycin and tylosin maintained the ability to induce the expression of *ermB* with GTG1:TAG mutations (Fig. 6I, J, K). In summary, we concluded that the translation of *ermBL2* is necessary for the induction of *ermB* expression by ery rather than Spi and Tyl. Additionally, GTG1: (TAA, TAG, TGA) mutations had different effects on the induction of *ermB* expression by Spi and Tyl, indicating that the RNA sequence itself, rather than the amino acid sequence of *ermBL2,* is critical for the induction of *ermB* expression by Spi and Tyl.

## Discussion

The slow discovery of new antibiotics and the emergence of a large number of antibiotic-resistant bacterial species have led to the possible use of some unpopular antibiotics. Macrolide antibiotics are used to treat infections caused by gram-positive and gram-negative bacteria [1]. These antibiotics have been effective in clinical use for over 70 years. Most of the macrolides currently used in the clinic are semisynthetic erythromycin derivatives composed of a 14- or 15-membered macrolactone ring. Therefore, there are a large number of drug-resistant bacterial species that are resistant to these antibiotics in the clinic. Because of the substantial clinical application of macrolides, they are not easily discarded. A simple strategy is to use macrolides that are not commonly used or not used in the clinic, such as 16-membered macrolides. We wanted to know whether 16-membered ring macrolides induce the expression of resistance genes similar to 14- or 15-membered ring macrolides and whether the mechanism of inducing expression is the same as that of 14- or 15-membered ring macrolides.

Four *erm* genes *(ermA, ermB, ermC, ermD)* whose expression is induced by erythromycin have been well studied [5, 7, 32]. Only the expression of *ermB* could be induced by 16-membered-ring macrolides in previous study [10, 21]. The *ermB* gene encodes the ribosomal methylase that dimethylates a single adenine in 23S rRNA, which leads to high macrolide resistance and bacterial survival [10, 11]. All the MLS_B_ antibiotics induced the expression of *ermB* [10]. This feature of *ermB* is different from other classes of erm (*ermA*, *ermC* and *ermD*), and the expression of *ermA*, *ermC* or *ermD* is induced by certain specific MLS_B_ antibiotics [7, 32]. In our reporter system, we found that 16-membered ring macrolides specifically induced the expression of *ermB* rather than *ermC*. Till date, there are three major mechanisms to control the expression of inducible macrolide resistance genes by different kinds of macrolide antibiotics. (1) Ribosome stalling on the leader peptide is the main mechanism to control the expression of inducible macrolide resistance genes, such as *ermAL* [32]*, ermBL* [6]*, ermCL* [7] and *ermDL* [5] induced by erythromycin. (2) Macrolide antibiotic-induced stabilization of resistance gene mRNA is another mechanism to control the expression of several inducible resistance genes [18, 22, 33]. (3) Regulation of *ermC* gene expression by ketolides is controlled by ribosomal frameshifting [34]. Translational attenuation and mRNA stabilization are mechanisms by which *ermB* expression is induced by erythromycin. The proposed translational attenuation model of the induction of *ermB* expression by erythromycin has been studied (Fig. S1) [17]. In the absence of erythromycin, *ermB* expression is repressed because the ribosome binding site 2 (GGAG) and AUG start codon of the *ermB* mRNA are sequestered in a stem–loop structure. An alternative stem–loop structure is changed in the presence of erythromycin, exposing the RBS2 and start codon of the *ermB* gene and causing the induction of *ermB* expression (Fig. S1).

The induction of *ermB* expression by 16-membered ring macrolides is not well documented. The purpose of this work was to investigate the mechanism by which 16-membered ring macrolides induce the expression of *ermB.* We used spiramycin and tylosin as standard 16-membered ring macrolides. We first constructed a reporter plasmid to sense antibiotics. Spiramycin and tylosin specifically induced the expression of *ermB* rather than *ermC*. In this study, introduction of a premature termination codon showed that the translation of the N-terminus of *ermBL* is necessary for the induction of *ermB* expression by Spi and Tyl, while the C-terminus of *ermBL* is not important for the induction of *ermB* expression by Spi and Tyl.

We found that Ery and 16-membered ring macrolides induced the expression of *ermB* via different mechanisms for the following reasons: (1) Ribosome stalling on *ermBL* at the tenth codon (Asp) is believed to be the major mechanism by which Ery induces *ermB* expression. We changed the tenth codon (Asp) to other amino acids and found that the majority of the mutations rendered the capacity of induction by 16-membered ring macrolides rather than erythromycin. (2) Gupta et al. revealed the ribosome as a highly selective sensor of Ery and telithromycin. Its ability to recognize and discriminate between Ery and telithromycin could be directly modulated by minor variations in the sequence of the nascent peptide [19]. If the mechanism by which 16-membered ring macrolides induce *ermB* expression are still relevant to this situation, then when we changed the other parts of the *ermB* regulatory region, Ery and 16-membered ring macrolides should show similar inducing effects. However, when we changed the length of *ermB*’ (N-terminal region of *ermB*), the induction effect was different between these two kinds of antibiotics (Fig. 3E), which means that the different mechanisms by which Ery and 16-membered ring macrolides induced *ermB* expression are not the same as the different mechanisms by which Ery and telithromycin induce *ermB* expression. (3) Alanine-mutational analyses have also shown that changes in the R7-D10 amino acids of *ermBL* had little influence on induction effects of Spi and Tyl, while gene expression induction by Ery was severely impaired. The latter part of *ermBL* is important for the induction of *ermB* expression by Ery and 16-membered ring macrolides. However, S22A and D23A had little influence on the induction effect of Spi and Tyl but severely impaired the induction effect of Ery (Fig. 4B). This further shows that the mechanisms by which these two kinds of antibiotics induce expression are different. (4) Furthermore, hybrid CL-BL or BL-CL constructions showed that the latter part of the *ermB* regulatory region is critical for the induction of *ermB* expression by Spi and Tyl.

*ErmBL2*, which exists in the *ermB* regulatory region, is critical for the induction of *ermB* expression by Ery [17]. The C-terminus of *ermBL* and the N-terminus of *ermBL*2 share base sequence but not amino acid sequence. *ermBL*2 is a (+ 1) frameshift compared with *ermBL* in the common area. We mutated the start codon (GTG) of *ermBL*2 to alanine, start codon or stop codon and found that the translation of *ermBL*2 is not important for the induction of *ermBL* expression by Spi and Tyl. GTG1:TAG mutation maintained the induction effect of Spi and Tyl, while the GTG1:TAA and GTG1:TGA mutations decreased the induction effect of Spi and Tyl, indicating that the RNA sequence itself, rather than the amino acid sequence, of *ermBL2* is critical for the induction of *ermB* expression by Spi and Tyl. GTG1: alanine (GCG, GCT, GCC, GCA) mutations also confirmed this conclusion.

Here, we used a well-studied *ermBL-ermB* operon (M11180) [16] *LacZa* fusion reporter plasmid as a model to investigate the detailed mechanism underlying expression induction by 16-membered ring macrolides [17, 18]. In summary, the translation of *ermBL* and the RNA sequence of the C-terminus of *ermBL* are critical for the induction of *ermB* expression by Spi and Tyl. The detailed mechanism needs further study, and the study of the mechanism underlying the drug resistance induced by 16-membered ring macrolides will be helpful for the treatment and prevention of the emergence of 16-membered ring macrolide-resistant strains.

## Conclusion

The translation of *ermBL* and the RNA sequence of the C-terminus of *ermBL* are critical for the induction of *ermB* expression by Spi and Tyl. The study of the mechanism underlying the drug resistance induced by 16-membered ring macrolides will be helpful for the treatment and prevention of the emergence of drug-resistant strains.

## Supplementary Information

**Additional file 1: Figure S1.** (A) Classical model of ermBL dependent regulation of ermB translation in the presence of erythromycin. (B) The second functional leader peptide named ermBL2 found in our previous work. **Figure S2.** (A)The detail sequence from begining of tac promoter to end of lacZa. (B) The detail sequence of ermB’ truncated mutations used in Figure 2E. **Figure S3.** The detail sequence of BL-CL and CL-BL constructions. **Figure S4.** Agar diffusion assays of the degree of induction by Ery in vivo following Ala mutation of ermBL amino acid sequences. **Table S1.** MIC determinations of E.Coli carrying the PGEX-ermBL-ermB’- lacZα plasmid.

## Data Availability

All documents and additional data are available from the corresponding author upon reasonable request.
