# Supplementary material for: 16-membered ring macrolides and erythromycin induce ermB expression by different mechanisms
Source: BMC Microbiol. 2022 Jun 9;22:152. doi: 10.1186/s12866-022-02565-3 (PMC9178857; doi:10.1186/s12866-022-02565-3)
Supplement: Supplementary file 1 — Additional file 1: Figure S1. (A) Classical model of ermBL dependent regulation of ermB translation in the presence of erythromycin. (B) The second functional leader peptide named ermBL2 found in our previous work. Figure S2. (A)The detail sequence from begining of tac promoter to end of lacZa. (B) The detail sequence of ermB’ truncated mutations used in Figure 2E. Figure S3. The detail sequence of BL-CL and CL-BL constructions. Figure S4. Agar diffusion assays of the degree of induction by Ery in vivo following Ala mutation of ermBL amino acid sequences. Table S1. MIC determinations of E.Coli carrying the PGEX-ermBL-ermB’- lacZα plasmid. [file 12866_2022_2565_MOESM1_ESM.pdf]

Figure S1

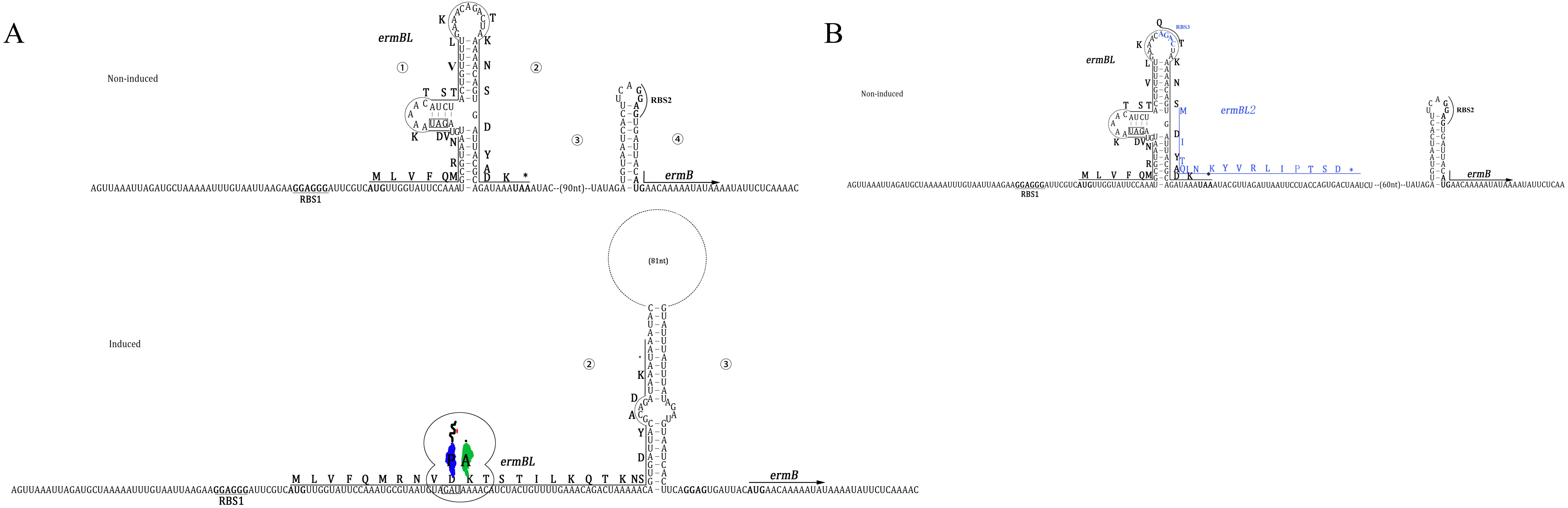

Figure S1. (A) Classical model of *ermBL* dependent regulation of *ermB* translation in the presence of erythromycin. (B) The second functional leader peptide named *ermBL2* found in our previous work.

A

tac promoter TGTTGACAATTAATCATCGGCTCGTATAATGTGTGGAATTGTGAGCGGATAAC
AATTGCTAGTCTAGAGAAGTTAAATTAGATGCTAAAAATTTGTAATTAAGAAG
ermBL GAGGGATTCGTCATGTTGGTATTCCAAATGCGTAATGTAGATAAAACATCTAC
ermBL2 TGTTTTGAAACAGACTAAAAACAGTGATTACGCAGATAAATAAATACGTTAG
ATTAATTCCTACCAGTGACTAATCTTATGACTTTTTAAACAGATAACTAAAATT
ACAAACAAATCGTTTAACTTCTGTATTTATTTATAGATGTAATCACTTCAGGAG
ermB' TGATTACATGAACAAAAATATAAAATATTCTCAAAACCTTAAGGATTCACTGG
CCGTCGTTTTACAACGTCGTGACTGGGAAAACCCTGGCGTTACCCAACTTAA
lacZa TCGCCTTGCAGCACATCCCCCTTTCGCCAGCTGGCGTAATAGCGAAGAGGCC
CGCACCGATCGCCCTTCCCAACAGTTGCGCAGCCTGAATGGCGAATGGCGCT
AACTCGAG

ermB regulatory region

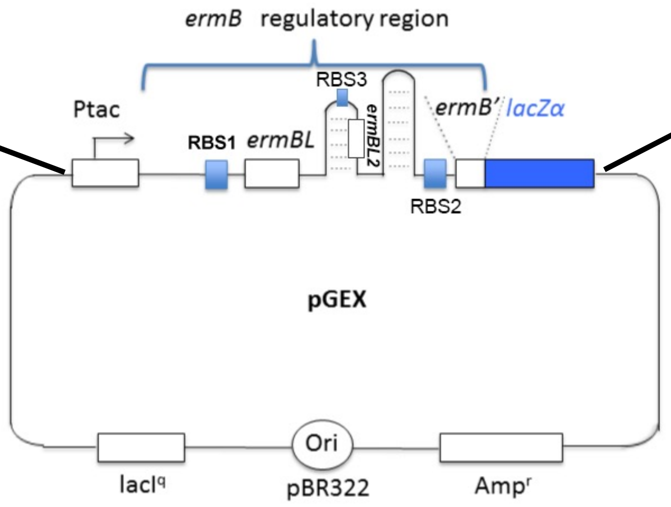

B

ermB'=M(ATG)

ermB'=MN(ATGAAC)

ermB'=MNK(ATGAACAAA)

ermB'=MNKN(ATGAACAAAAAT)

ermB'=MNKNIKYSQN(ATGAACAAAAATATAAAATATTCTCAAAAC)

Figure S2. (A)The detail sequence from begining of tac promoter to end of *lacZa*. (B) The detail sequence of *ermB'* truncated mutations used in Figure 2E.

ErmB Regulatory region

GAAGTTAAATTAGATGCTAAAAATTTGTAATTAAGAAGGAGGGATTCGTCATGTTGGTATTCCAAATGCG
TAATGTAGATAAAACATCTACTGTTTTGAAACAGACTAAAAACAGTGATTACGCAGATAAATAAATACGT
TAGATTAATTCCTACCAGTGACTAATCTTATGACTTTTTAAACAGATAACTAAAATTACAAACAAATCGTT
TAACTTCTGTATTTATTTATAGATGTAATCACTTCAGGAGTGATTACATGAACAAAAATATAAAATATTCTC
AAAAC

ErmC Regulatory region

attttataaggaggaaaaaatatgggcatttttagtatttttgtaatcagcacagttcattatcaaccaaacaaaaaataagtggttataatgaatcgttaataagcaaaattcat
ataaccaaattaaagagggttataatgaacgaga aaaatataaaacacagtcaa

BL-CL

GAAGTTAAATTAGATGCTAAAAATTTGTAATTAAGAAGGAGGGATTCGTCATGTTGGTATTCCAAATGCG
TAATGTAGATAAAacagttcattatcaaccaaacaaaaaataagtggttataatgaatcgttaataagcaaaattcatataaccaaattaaagagggttataatga
acgaga aaaatataaaacacagtcaa

CL-BL

attttataaggaggaaaaaatatgggcatttttagtatttttgtaatcagcACATCTACTGTTTTGAAACAGACTAAAAACAGTGATTA
CGCAGATAAATAAATACGTTAGATTAATTCCTACCAGTGACTAATCTTATGACTTTTTAAACAGATAACT
AAAATTACAAACAAATCGTTTAACTTCTGTATTTATTTATAGATGTAATCACTTCAGGAGTGATTACATG
AACAAAAATATAAAATATTCTCAAAAC

Figure S3. The detail sequence of BL-CL and CL-BL constructions.

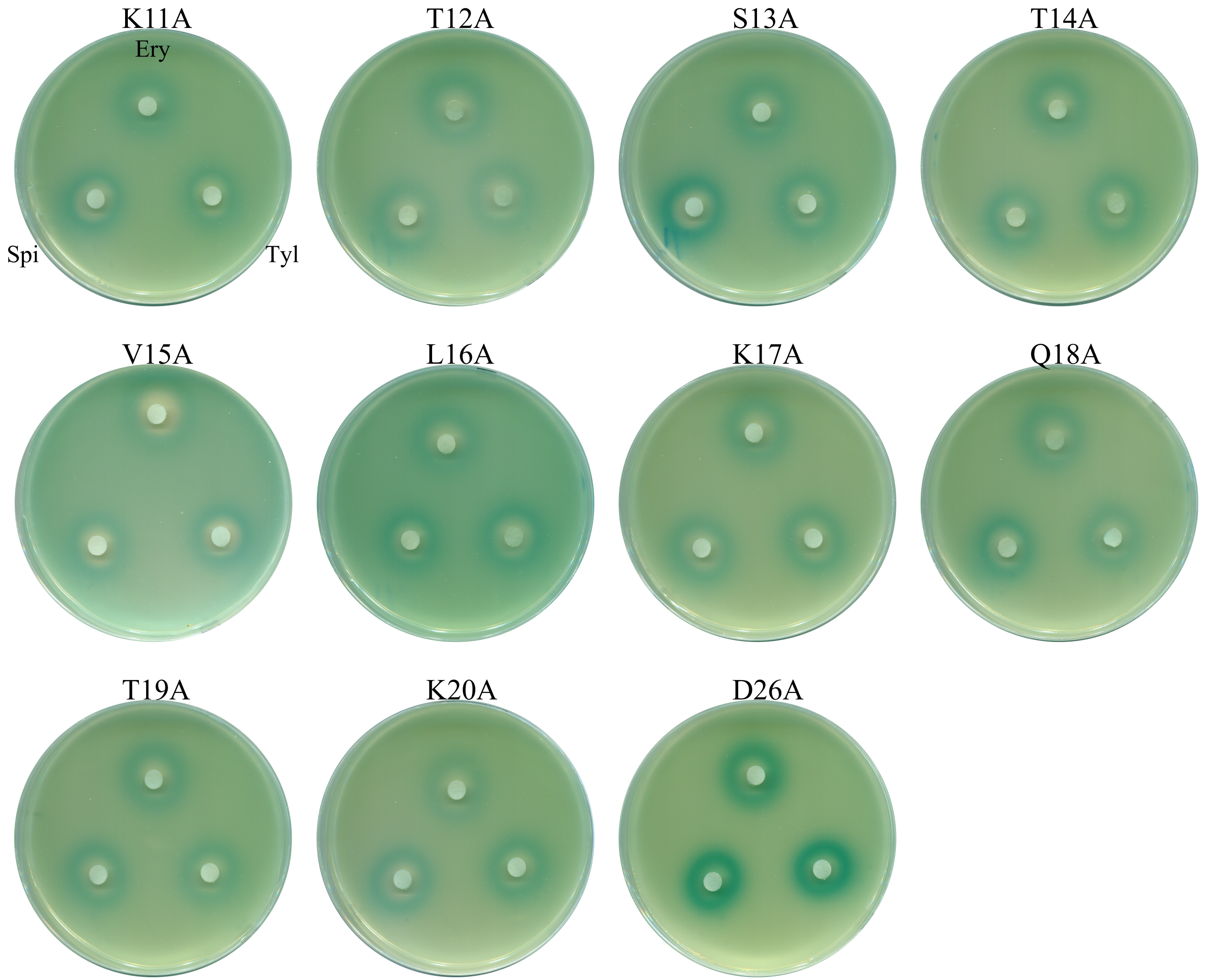

Figure S4. Agar diffusion assays of the degree of induction by Ery *in vivo* following Ala mutation of *ermBL* amino acid sequences

Table S1. MIC determinations of *E.Coli* carrying the PGEX-*ermBL-ermB'- lacZα* plasmid

Antibiotics	MIC(μg/ml)
Erythromycin	1024
Spiramycin	4096
Tylosin	4096

MIC determinations

Minimal inhibitory concentration (MIC) determinations were carried out as described(1, 2). The method has been slightly modified from the reference mentioned. Briefly, *E. coli* strain JM109 containing pGEX-*ermBL-ermB'-lacZ*α plasmid was grown overnight in LB medium containing 100 μg/mL of ampicillin. The next day, dilute cells 100-fold into fresh LB medium containing ampicillin and were grown for 2h. Exponential-phase cultures were then diluted to an A600 (OD_{600}) of 0.002 and placed into fresh LB medium with 3 ml/tube. After the addition of antibiotics (0, 2, 4, 8, 16, ……,1024, 2048 and 4096 μg/mL), the tubes were incubated for 15h at 37°C without shaking following incubation for 3h with shaking. The MIC was recorded as the lowest concentration of drug in the tube with no obvious turbidity.

1. Xiong L, Korkhin Y, Mankin AS. Binding site of the bridged macrolides in the Escherichia coli ribosome. Antimicrob Agents Chemother. 2005;49(1):281-8.
2. Wang S, Jiang K, Du X, Lu Y, Liao L, He Z, et al. Translational Attenuation Mechanism of ErmB Induction by Erythromycin Is Dependent on Two Leader Peptides. Front Microbiol. 2021;12:690744.
